# The State of Lunacy in Ireland

**Published:** 1862-01

**Authors:** 


### Art. VI.—THE STATE OF LUNACY IN IRELAND.

The Tenth Report, to His Excellency the Lord Lieutenant, on the District, Criminal, and Private Lunatic Asylums of Ireland now lies before us, and from it we gather the following particulars illustrative of the state of lunacy in that division of the kingdom during the two years ending the 31st of March, 1861.

The old asylum buildings, erected thirty years ago, are gradually, it would appear, losing much of their original gloominess and prison-like appearance; while modern improvements, tending to the comfort, well-being, and efficient treatment of the patients in them, have been introduced, and are in process of being so. The efforts of the Inspectors of Lunacy for the further amelioration of the condition of the insane classes of the country, it is satisfactory to learn, have been met in an excellent spirit, and generally acted upon by the local authorities; and every disposition is manifested by the gentry and community to provide suitable accommodation for the lunatic poor, "now that they are satisfied of the utility thereof, and made acquainted, not alone with the extent to which insanity exists throughout the country, but with the social dangers and disadvantages that are likely to ensue unless active and prompt steps be taken to counteract its progress."

It is worthy of note, that a like knowledge of the extent and dangers of lunacy does not exist among the gentry and community of this country. Is it not probable that this may be one, and a serious, source of the difficulties too commonly thrown in the way of a sufficient provision for our pauper lunatics?

The Inspectors tell us that they have not failed to express their conviction of the necessity of having ample accommodation for the insane poor, when bringing the subject under the notice of the Boards of Governors of District Asylums. They have also "attended on Grand Juries for the purpose of placing before them the requirements of their different counties, as shown by statistical returns, as well as the advantages likely to be derived from the breaking up of large districts, or those where two or three counties had but one asylum between them. In fact, their object so far has been to establish the general principle, that where a county comprises a very extensive area, or contains a population bordering on 200,000, a separate asylum would prove the most advantageous. The greater expense to be incurred by the erection of a new institution as compared with that of enlarging an old one, and the consequent employment of a second staff, constituted, in the opinion of some parties, the main objection to this principle; it being agreed, on all hands, that additional accommodation of some kind was required, as at Carlow, Derry, Limerick, and elsewhere. Differences arose, however, as to whether it would be more advisable that fresh districts should be formed, or existing asylums enlarged." The point in dispute was submitted to the Lord-Lieutenant, who decided that fresh districts should be formed. In consequence, Orders in Council have been issued for the erection of an asylum in each of the following counties: Wexford, Clare, Mayo, Donegal, and Monaghan.

The Inspectors pass in review the chief alterations suggested or required in existing District Asylums; and speaking of the Cork Asylum, they refer, in the following terms, to the introduction of the Turkish Bath there, at the suggestion of the Resident Physician:—

"So far as we can form an opinion from the cases submitted to us by Dr. Power, who employs it on an average number of from fifteen to twenty cases daily, we have reason to hope, without attributing any direct curative effects to its use, that it will prove a beneficial agent. It has been found to exercise a very soothing influence, and the patients themselves are reported as by no means adverse to its employment. Considering that the simple hot bath has long been regarded as a valuable adjunct in the treatment of mental disease, it is but reasonable to infer that the Turkish would produce more marked results. In proportion, however, to its powers, caution should be observed in its use. The cost was about 180*l.*, including fitments, by which a portion of the heat generated in it, and which otherwise would be wasted, is rendered available for drying-closets and other domestic purposes."

If the asylum-extension now in progress, or about to be undertaken in Ireland, be coupled with the existing accommodation, it would appear that the asylum provision of the sister island is equal to, if not decidedly above, that of any other country in Europe. There is existing accommodation, provided by public contribution, in the district asylums, for 4500 lunatic poor, and the additional accommodation in progress will raise this number to 6400—the aggregate population of the country being less than six millions. Fourteen years ago, when the population amounted to over seven millions, provision was made for only 2600 lunatic poor.

The average cost of maintenance of each patient is estimated at 20*l.* a-year. The annual cost of 6400 lunatic patients would, therefore, amount to 128,000*l.*, "which, levied on the Grand Jury valuation, will be as nearly as possible 2½*d.* in the pound —a levy, however, independent of that for the erection of buildings and the purchase of land, which, advanced in the first instance by Government, is repayable in fourteen years, the original sum not bearing interest."

The *results of treatment* during the two years the report embraces, were not quite so satisfactory as in the preceding biennial period, but still they place Irish asylums, so far as their statistics show, in the first rank in this respect. The cures calculated on admissions have been 46·64 as against 48·71 in the two previous years; or, taking the daily average of patients, as 14·86 against 16. The mortality, principally from pulmonary and cerebral affections, amounted to 705, or a total of 10·740 under treatment, constituting a per centage of only 6·56. Coroners' inquests were held on ten deaths looked upon as being sudden or accidental, and in one instance only was culpability from negligence recorded against the attendant in charge. The inspectors state that the management of the District Asylums is, on the whole, conducted in a very satisfactory manner.

The advantages derived by lunatics from the regular attendance of chaplains are referred to in high terms. "In fact," say the inspectors, "we believe that in no institutions do the outward ministrations of religion produce a more humanizing effect than in those for the insane. It is, therefore," they add, "with deep concern we have to observe that in one asylum alone in the three kingdoms—that at Belfast—the majority of a most influential, moral, and highly enlightened [?] board, deny admission with the means of carrying on Divine worship, to the clergymen duly appointed for that desirable purpose."

Not only are the patients in the District Asylums industriously employed both in and out of doors, and their sanitary comforts attended to, but their amusement also is not neglected. The Richmond, Sligo, Belfast, and other asylums, have their regularly trained bands of music recruited from the patients, and apartments specially set aside for the purpose of recreation. The first-named asylum (the Metropolitan) is, moreover, transformed into a complete educational establishment, competent instructors being engaged to teach the inmates reading, writing, arithmetic, singing, &c. Indeed, the Inspectors propose to seek assistance, through a grant of books and other tuitional requisites, from the Board of National Education, for those asylums in which the number of patients will justify the application. Much as may be said in favour of the present state of the Irish asylums, it is manifest that even a better lot still is in store, thanks in an especial manner to the energy and efficiency of the Lunacy Inspectors.

There are certain peculiarities in the social condition of the Irish insane poor which, however, it is important to bear in mind, in instituting any comparisons between the Irish and English or Scotch asylums, or in estimating the requisites for the former. This is well set forth in the Report. It is there observed by the Inspectors:—

" ——Desirous as we are to promote in every way the amusement of the inmates of our District Hospitals, and to afford them every means of in and out-door occupation suited to their antecedents, it should not be forgotten that a large proportion of them is composed of agricultural labourers, and of individuals from the humblest walks in life. In England, on the contrary, while the insane of a similar position are located to a considerable extent in licensed houses, or, for the sake of economy, in the lunatic wards of union buildings, the regularly constructed borough and county asylums, erected at a cost fully one-third greater than ours, are peopled, for the most part, from the artisan and better instructed classes, and from the shopkeeping and farming communities—both alike accustomed to many domestic comforts, and to which the rural population of this country, it must be admitted, are strangers in no small degree. Nevertheless, although for all practical purposes there may exist an equal amount of substantial advantages in Irish asylums, as shown in the average scale of dietary, clothing, &c., which obtains in them, as well as in the attention paid to direct personal necessities, remedial and other, but above all, in an undeviating humanity towards the insane, it should not be a matter of surprise if, considering the relative social condition of their inmates, a deficiency of furniture, carpeting, and ornament is noticeable in them, as contrasted with many of the more expensively supported establishments in the sister kingdom. Comparisons having occasionally been made between these similar institutions of either country, with reference principally to their interior arrangements, and wishing to uphold, but in a spirit of candid emulation, the character and usefulness of our own, we have entered on these explanatory remarks; and would further state for your Excellency's satisfaction that, while many of our institutions are kept with a most creditable taste and neatness, no longer presenting that nakedness hitherto so much complained of *in all*, under the careful superintendence of their Resident Medical Officers, order, regularity, and a regard to outward comforts, are steadily progressing from day to day, with an extension of certain improvements originated in this, and which are being adopted in other countries."

The Report touches very briefly on the misunderstanding between the Resident and Visiting Physicians at the Maryborough Asylum, which has recently given rise to much acrimonious discussion in the public press, deputations to the powers that be, and so forth, on the status of Resident Physicians. Concerning the specific misunderstanding, the remark of the Chief Secretary on the absence "of forbearance and consideration for the feelings of others" is cited, and the Inspectors express a hope that the late Commission of Inquiry into the matter, together with the new rules passed in council, will bring round a more agreeable state of things. They reiterate, also, the opinion expressed in their Ninth Report, on the expediency of having Consulting or Visiting Physicians attached to District Asylums, on the ground especially of the sense of security which their appointment affords the public at large, but they add:—

"— We are not the less satisfied that the status of the Resident Medical Superintendents, who, as a body, are devoted to their duties, ought to be distinctly recognised, and as being directly responsible for the well-being of the institutions intrusted to their care, that their managerial position should be upheld intact. For these desirable objects, among others, a new code of rules and regulations are being prepared after mature deliberation, for, we hope, the more cordial and efficient management of Irish District Asylums, and which, if sanctioned by your Excellency in Council, will have the full force of a statute."

The average annual cost of maintenance of each patient during the two years amounted to 20*l.* 5*s.* 7*d.*, as against 19*l.* 19*s.* 2*d.* in the two preceding years. A comparative estimate of the cost of maintaining lunatics in poor-houses, gaols, and asylums, shows that the difference is apparently greater between the former and the latter than in England—the maintenance in a poor-house being estimated at 13*l.* per head. The Inspectors, however, very properly intimate, that somewhat higher motives than mere pecuniary economy weigh heavily in favour of asylums.

The erection of District Asylums in Ireland, at first confided to Commissioners especially appointed by the Government for the purpose, and subsequently vested in the Board of Public Works, has recently been transferred (by virtue of an Act of Parliament) to a Board of Control. This board, first appointed in 1860, consists of four Commissioners, two being the Inspectors of Lunacy.

The number of lunatics in *workhouses* had increased from 2047 individuals on the 1st of April, 1859, to 2534 on the 31st of March, 1861. The confinement of lunatics in these institutions is open to the same objections in Ireland as in England. When, in 1859, the subject of increased accommodation for lunatics in certain districts was under consideration, it was suggested that some union buildings, then almost untenanted, from the decrease of pauperism in the districts, might, perhaps, be converted into lunatic asylums at a considerable saving of expense. Estimates were prepared, in one or two instances, to test the feasibility of this suggestion, when it was found that the cost would equal two-thirds of that required to build a new asylum, while the arrangements would be of an inferior character. The suggestion was therefore set aside by the Executive.

No less than 1365 dangerous lunatics were committed to *gaol* within the two years—a striking illustration of the existing insufficiency of asylum accommodation. "In some districts the prisons may be almost regarded as subsidiary asylums."

The patients in the *Central Criminal Asylum* at Dundrum number 133.

"Within the last two years [the Report states] thirty patients have been admitted to, and seventeen discharged from it. Of the latter, three were liberated, on their recovery, through the clemency of the Lord Lieutenant; seven were discharged as sane, on the expiration of their sentence; three were remitted, cured, to convict prisons, and five were sent by the Inspectors, at the end of the legal period of confinement, to the asylums of the districts to which they appeared to belong. Thus of the total discharged, twelve had been restored to reason."

The practice of the Inspectors in the discharge of criminal lunatics is thus stated:—

"As a rule, when the offence committed has been of an aggravated or serious nature against the person, a full year's probation of sanity, with a certificate of undeviating good character, is required by us prior to forwarding any favourable report of the party. When also there is a reasonable doubt as to the mental condition of the offender at the period of the commission of the offence, or where he has evinced no genuine symptoms of lunacy after its perpetration—

furthermore, should a strong and anything like a justifiable prejudice exist in the public mind, or should personal apprehensions be harboured by the injured party (and such instances are not rare), we are most unwilling to interfere. At the present moment, besides others, there are three homicides quite rational, who have been so for a considerable time—we might even say since their reception—but on whose behalf we have as yet taken no steps. Increased experience continuing to impress us with a belief, the expression of which we have already hazarded, that the act of the lunatic in nine cases out of ten, let it eventuate even in murder, is perhaps more a symptom or the result of disease than aught besides—the casual product of some passing excitement or strong delusion—when a party becomes thoroughly sane and continues so for a lengthened period, unless there be special reasons to the reverse, we feel justified in recommending the exercise of clemency; and, therefore, do not yield to any abstract prejudice hostile to the liberation of a recovered criminal lunatic whose disposition and behaviour, brought continually under our observation, have given satisfaction to the Resident Physician, Dr. Corbet, and whose sanity has been conjointly certified by him and the Visiting Physician. One fact is certain, namely, that some of the best behaved and, in disposition, the kindliest of those in the Criminal Asylum at Dundrum, have unfortunately deprived some of their fellow-beings of life. We do not mean, however, to ignore the existence of strong homicidal propensities among the insane, and combined with them a most dangerous and studied adaptation of the means to the end—greater perhaps than exists in connexion with any other delusion of the human mind.

"Since the opening of the Central Asylum, the number of patients who have obtained free discharges amount to twenty-eight; and it is gratifying to us to be able to state that, up to this date, there has not been, as far as we are aware of, any instance of misconduct on the part of the persons so discharged."

The Report speaks favourably of the majority of the *private licensed houses;* and although one or two omissions of due attention to the requirements of the law are noted, "no abuse," it is said, "or tangible act of harshness," has come to the knowledge of the Inspectors. Shameful neglect, nay, desertion, by families, of lunatic relatives placed in asylums, is of by far too frequent occurrence in Ireland as in England. It seems to be thought by many that every moral and social obligation has been fulfilled if a lunatic relative has been once safely lodged in an asylum. Four new houses have been licensed in the two years. "As exemplifying the longevity of lunatics when properly cared for," say the Inspectors, speaking of the Government Asylum (containing eighty-six patients) at Lucan, "these parties will be found to average between sixty and seventy years of age, and, one with another, are nearly thirty years in hospital. The mortality among them has been singularly low for some years past, being under four per cent."

The Inspectors remark upon the want of an asylum of the class that has been designated with us "middle-class"—that is, which would offer accommodation for those whose means will not enable them to pay the ordinary expenses of a private asylum, and yet debar them from entering a public one.

*Is insanity on the increase?* To this question the Inspectors reply much in the same fashion as the English Commissioners of Lunacy have answered it, *apropos* of England, in their last report.—

"We have no certain facts [they say] to guide us to a certain conclusion as to whether mental diseases are in a state of greater propagation now than formerly. We know, however, and it appears to us a natural result, that with fresh accommodation for the insane, fresh though long existing cases are presented for admission into asylums, and thus an apparent increase of lunacy may be observed. Other causes have been combining to add directly (without an actual extension of the malady itself) to a perceptible growth, or an enlarged ratio of the insane to the sane part of the population.

"1st.—The care bestowed on the lunatic classes, and the improved system of treatment latterly adopted in their regard, have tended materially to the prolongation of life among them, and to their consequent accumulation. Of this fact, instances are afforded by the patients at the Lucan Spa House; their ages averaging from sixty to seventy years—the disease in some parties existing over thirty.

"2ndly.—Science, and even public opinion, now accept as indicative of lunacy, affections which in former days, though then quite as much developed as in the present, were classed under a different category from mental disease or aberrations of the intellect; and though it may occasionally happen, particularly in the case of criminals, that a forced extension is made of insanity to protect the accused, there can be no doubt that in numerous instances punishment formerly awaited on offences to which, judged by the present recognised symptoms of mental alienation, it would now be deemed a just and legitimate bar.

"3rdly.—Another reason which gives a greater show to insanity now-a-days is connected with the gradually and happily expiring sentiment, that lunacy is as it were a discreditable visitation to the families of the afflicted—a sentiment not ignored formerly even by the humblest class of life. Formerly patients were frequently kept concealed in garrets among the rich, in out-houses among the poor, till liberated by death. Fortunately this feeling is all but extinct, and asylums, both public and private, are now gladly taken advantage of when the necessity unhappily arises.

"4thly.—In this country emigration, while it has so seriously diminished the population at large, has not in a like manner affected the number of lunatics, the majority of whom, left behind, have not only found a refuge in our public institutions, but, protected in them from all the sufferings and privations incidental to the poor, are ensured a greater likelihood of a longer existence. Thus, then, though in reality at the present date and thirty years ago the relative proportion of the insane to the sane born in Ireland might be nearly equal, a far greater amount of lunacy is now recognizable. There exists, however, little doubt but that insanity is, in great measure, a disease of intellect—one connected with the development of the human mind; and it is therefore highly presumable that in this age of excitement and rapid advancement in arts and sciences mental affections may be more prevalent than before; but in the absence of statistical data, as yet unobtained by us, the extent thereof can be but a mere matter of surmise."

The Inspectors note that during the two months that *religious revivalism* was prevalent, a year or two ago, in the northern district of the island, more cases of insanity resulted therefrom than had taken place in the whole preceding year.

"It would be a mistake, however," they add, "to suppose that because the immediate cause of mental aberrations may be referrible to religious enthusiasm, the type of the disease, or the delusions themselves, must be of the same character; the reverse would almost seem to hold good, as proved by the number of parties committed at the time to gaol as violent or dangerous lunatics. Many persons with a predisposition to the malady would pass through life quite sane in all its relations, if not yielding to dissipation, or exposed to strong exciting causes, which, no matter what they be, will develop the weaker points and latent tendencies of the mind."

The Report terminates with an analysis of the valuable Statistical Appendix, which as usual accompanies it, and from which we derive the following particulars:—

The number of reputed lunatics, idiots, imbeciles, and epileptics existing in Ireland, exclusive of those confined in asylums, gaols, and workhouses, amounted on the 1st April, 1861, to 8991, of whom 4959 were *males* and 4032 *females*. These individuals were classified in the following manner:—*Lunatics*, 3 48 (males 866, females 785); *idiots* and *imbeciles*, 5469 (males 3148, females 2321); *epileptics*, 1871 (males 945, females 926).

The returns from which the foregoing numbers are derived were obtained through the police, no class of the community being exempted from the inquiry, rich and poor being alike included; and they indicate an increase of 1379 upon similar returns obtained in 1856. The returns "merely show, or profess to show," the Inspectors remark, "the total number of persons reputed to be more or less mentally affected, or, in other words, the stock from which lunacy may be engendered;" but they add, "we are induced to think that these returns . . . . are approximatively correct, from the fact of the relative proportion of the whole of the sexes with each other; the males being 4959, and the females 4032—the difference in excess of the former being fairly referrible to the greater number of wandering idiots to be found among them."

The number of lunatics, idiots, and epileptics in the *union workhouses* of Ireland on the 31st of March, 1861, was 2534, 965 being *males* and 1569 *females.* The *lunatics* amounted to 635 (190 males and 445 females); *idiots* and *imbeciles,* 1020 (468 males, 552 females); and *epileptics* 879 (307 males and 572 females)."

The admissions into *district asylums* during the two years amounted to 2575, and the total number of patients in asylums during the year ending 31st March, 1861, was 5437. The absolute recoveries in the two years were 1201, and 298 were discharged improved, and 127 unimproved, on the requirement of their friends. "The deaths on the average number under treatment, 8411, were 705, of which ten were accidental and suicidal. Analysing these figures, the proportion of recoveries on admissions would appear to be 46·64 per cent. and on the daily average under treatment 14·27, while the percentage of death, calculated on the latter basis, amounts to only 8·38. It would appear that of the admissions, 478 were relapsed cases, but, it may be observed, not necessarily within the biennial period; for one of the characteristics of mental disease is its recurrent nature, even after many years of perfect health." Of the 2575 cases admitted, only fourteen were under ten years of age, and these mostly idiotic children who had evinced dangerous tendencies. The great majority, namely, 788, were between the ages of twenty and thirty; 553 were from thirty to forty; and the numbers in each successive decade from that period diminish gradually, only twenty-eight being admitted over seventy years of age.

From the tables showing the duration of the disease prior to admission into the asylum, and the periods of detention there, we learn, that in 60·11 per cent. (722 cases) of the total number of recoveries, asylum treatment had been had recourse to *within four months from the appearance of the disease.* Further, the chances of recovery, it would seem, are most between the first and fourth month after admission, "the cases during that period being 506 against 399 during the succeeding eight months, and so on in the inverse ratio as to time."

The proportion of the probably curable to the probably incurable stands at 1073 to 3216.

The tables narrating the supposed *causes of insanity* show that moral causes predominate with regard to females, physical with regard to males. In 800 ascertained cases, moral influences are noted as the exciting cause in 532 females and 368 males, while in 505 males the disease is ascribed to physical influences, but only in 286 females. Intemperance and irregularity of life are the assigned causes in no less than 241 of the male cases, but in 82 alone of the female cases. Hereditary transmission was traceable in 495 cases out of 2186; and 37 per cent. (818) of the whole number were attributed to hereditary transmission and intemperance combined. "The most melancholy instance of this," say the Inspectors, "that has come under our notice was in a northern workhouse, in which were four imbecile or epileptic children, the offspring of parents, one of whom had died insane, and the other from the effects of *delirium tremens.*" They add, "With reference to the hereditary character of insanity, we would observe that mental, like bodily affections, gradually wear out from the intermixture of blood; we have not seen a case in unbroken descent to the fourth generation; the most marked instance we know at present is in the Carlow Asylum, where the grandfather and father had both been patients, the latter dying in it; the son is now an inmate. The spread of lunacy would appear to be more through a collateral extension within certain generations."

The following figures indicate the *social condition* of the patients in Asylums—the numbers of *males* being 2165, of *females* 2124. The *married* amount to 983 (429 males and 554 females); *unmarried* 2914 (1297 males and 1617 females); *widowed* 272 (68 males, 204 females). "The explanation of this discrepancy [in the respective proportions between marriage and celibacy and the different sexes]," the Inspectors remark, "exists, we believe, in the fact that the moral causes of insanity, which find their origin in the griefs and anxieties incidental to married life, press less heavily on man than on the sensitive mind of woman; while as regards widowhood, the woman, deprived of her natural support, and thrown perhaps on the world, must feel her position more acutely than a man correspondingly situated. In married life, it may also be added, the protection of home exercises a salutary influence on the conduct of the male sex, whilst among unmarried men, on the contrary, continued dissipation in early life, irregular hours, and among other causes we are disposed to enumerate the immoderate use of tobacco (so injurious to the nervous system), act as predisposing causes to mental affections."

Finally, the *educational condition* of the patients in Irish asylums may be gathered from the facts that out of 4289 individuals, 266 only are well educated, 625 read and write well, and 974 read and write indifferently; thus it is to be inferred that 56 per cent. are uneducated. "In one institution [say the Inspectors] belonging to an agricultural district and containing 333 inmates, 36 alone can read and write well; while in another large asylum, connected with an important manufacturing city in the north, with 357 patients taken from a mixed community, the small number of 19 represents the fairly educated; facts fully bearing out the remark already made by us with regard to the social condition of the classes from which public asylums in Ireland, are mainly peopled."

---

